# Local TV News Coverage of Racial Disparities in COVID-19 During the First Wave of the Pandemic, March–June 2020

**DOI:** 10.1007/s12552-022-09372-5

**Published:** 2022-07-15

**Authors:** Yiwei Xu, Elizabeth K. Farkouh, Caroline A. Dunetz, Sravya L. Varanasi, Sophia Mathews, Sarah E. Gollust, Erika Franklin Fowler, Steven Moore, Neil A. Lewis, Jeff Niederdeppe

**Affiliations:** 1 Cornell University, Ithaca, NY 14853 USA; 2 Mayo Clinic Alix School of Medicine, Rochester, MN 55905 USA; 3 University of Minnesota, Minneapolis, MN 55455 USA; 4 Wesleyan University, Middletown, CT 06459 USA

**Keywords:** Racial/ethnic health disparities, Social determinants of health, COVID-19 pandemic, Local television news, Content analysis

## Abstract

The COVID-19 pandemic has disproportionately impacted health and social outcomes for people of color in the United States. This study examined how local TV news stories attributed causes and solutions for COVID-19-related racial health and social disparities, and whether coverage of such disparities changed after George Floyd’s murder, during the first wave of the COVID-19 pandemic. We systematically validated keywords to extract relevant news content and conducted a content analysis of 169 discrete local TV news stories aired between March and June 2020 from 80 broadcast networks within 22 purposefully selected media markets. We found that social determinants of COVID-19 related racial disparities have been part of the discussion in local TV news, but racism as a public health crisis was rarely mentioned. Coverage of racial disparities focused far more attention on physical health outcomes than broader social impacts. Stories cited more structural factors than individual factors, as causes of these disparities. After the murder of George Floyd, stories were more likely to mention Black and Latinx people than other populations impacted by COVID-19. Only 9% of local news stories referenced racism, and stories referenced politicians more frequently than public health experts.

## Introduction

The COVID-19 pandemic has disproportionately impacted health and social outcomes for people of color in the United States. During the early stage of the pandemic, the U.S. Centers for Disease Control and Prevention (CDC) reported that Latinx, Black, and Indigenous people experienced a higher likelihood of infection, severe illness, hospitalizations, and death due to COVID-19 (Moore et al., 2020; Romano et al., 2021). People of color have also faced more burdensome societal outcomes like increased food insecurity among Black people (Dubowitz et al., 2021), pandemic-induced housing insecurity among Black and Latinx people (Benfer et al., 2021), and increased hate crimes/bias incidents toward Asian people (Le et al., 2020).

There are several reasons why Latinx, Black, Indigenous, and people of color are more likely to face negative health outcomes (e.g., disease mortality) than white people. Structural or societal level factors (i.e., social determinants of health) are the main causes of health and social inequities, not individual decisions about health behaviors (Tipirneni, 2021; Williams & Jackson, 2005). These structural level factors, such as inequalities in social, economic, and environmental conditions, can affect disease outcomes through various mechanisms, including less access to social, medical, informational, and economic resources, racial discrimination, and resulting physiological stress responses (Link & Phelan, 1995). When it comes to COVID-19, the most important causes of inequity have been broad social inequality stemming from economic, labor, insurance, and housing policies, leading to racial and ethnic group differences in health care access, number and severity of pre-existing medical conditions, and vulnerability to environmental impacts (Tipirneni, 2021). Furthermore, many historically marginalized racial and ethnic groups work in roles deemed as essential workers, and due to economic and housing precarity some live with multiple generations of families, both of which can exacerbate COVID-19 impacts (Moore et al., 2020). More specifically, many Indigenous communities, due to centuries of displacement, oppression, and marginalization, have chronically underfunded infrastructure and thus suffer from a lack of access to basic needs. Several factors like small dwellings, multigenerational living, and lack of access to preventive measures (e.g., clean water, soap, and disinfectant) all contribute to such health disparities related to COVID-19 (Curtice & Choo, 2020; Wang, 2021). Even though individual behaviors, such as mask wearing, hand hygiene, and social distancing, are important to COVID-19 prevention (Chiu et al., 2020), social determinants of health are the primary drivers of COVID-19 related racial disparities.

### Media Framing and Racial Health Disparities

News media coverage and framing of health disparities can influence the ways that people think about COVID-19-related racial disparities. We adopted a broad definition of media framing as “the words, images, phrases, and presentation styles that a speaker uses when relaying information about an issue or an event to an audience” (Chong & Druckman, 2007, p. 100). By analyzing news media framing, researchers can understand how many members of the public are exposed to interpretations of data and events. The news media’s construction of events through visual and narrative aspects of a story further invites audience members to interpret and understand them in a certain way. Given limitations of time and space, stories that emphasize some aspects of a story at the expense of others further influence audience perceptions of how salient or important these considerations are (Entman, 1991). While the literature on news media framing has many subcategories, we focus here on emphasis framing, which explores “a problem definition, causal interpretation, moral evaluation, and/or treatment recommendation” (Entman, 1993, p. 52). Emphasis framing involves many different noteworthy elements of journalistic storytelling, including how stories attribute responsibility, whether they emphasize drama versus substance, and the extent to which they employ statistical data to convey broader trends.

In this study, we examine attribution and responsibility framing, which refers to how a news story assigns blame or culpability for an issue (i.e., causal responsibility) and/or solutions it offers for that issue (i.e., treatment/solution responsibility) (Iyengar, 1994). In the context of COVID-19-related racial disparities, attribution and responsibility frames deliver information to the audience about potential causes of, and solutions for, such health disparities. Attribution and responsibility frames related to health disparities can influence the ways that audiences attribute the causes and solutions of COVID-19-related racial disparities. Studies have found that when a social issue is framed as a general societal problem (i.e., thematic framing), audiences tend to attribute the problem to the government; when the same issue is framed as a personal instance (i.e., episodic framing), audiences tend to assign the responsibility to individuals themselves (Iyengar, 1990, 1994). Researchers have also found that news stories featuring personal anecdotes that emphasize social, structural, and/or environmental causes and solutions for social problems can increase audiences’ societal (versus individual) cause attributions about a health problem (Niederdeppe et al., 2014).

Despite well-established structural explanations for racial health disparities, previous research on news coverage has found that individual behavioral explanations (versus structural explanations) dominate discourse of racial/ethnic health disparities in the U.S., which could limit public support for policy solutions addressing racial health disparities (Kim et al., 2010; Nagler et al., 2016; Taylor-Clark et al., 2007). Others have argued that health advocates should frame messages to emphasize the role of social determinants of health in shaping health outcomes, rather than emphasizing individual behavioral decisions, to raise awareness of health disparities and generate support for policies with potential to ameliorate them (Niederdeppe et al., 2008).

In this study, we examine local TV news media, which is the most watched news outlet and an important source for Americans to obtain health-related information (Gollust et al., 2019; Pew Research Center, 2021). For COVID-19 related information, people trust local TV news and network news more than most other forms of news media (Lopes & Stokes, 2021). This highlights the need to understand when and how local TV news covered COVID-19-related racial disparities, with particular attention to how news media used attribution and responsibility frames to cover COVID-19-related racial disparities.

### Previous Analyses of COVID-19-Related News Coverage and Racial Health Disparities

To date, however, few studies have examined how the news media, and local TV news in particular, have covered racial/ethnic health disparities related to the COVID-19 pandemic. COVID-19 related news coverage increased substantially in March 2020 and declined gradually thereafter in May and June (Mach et al., 2021). One study found that national online newspaper outlets (January to March 2020) most commonly emphasized the financial impact of COVID-19 (11.6%), stories of individuals affected by COVID-19 (7.0%), COVID-19 death and death rates (6.8%), precautionary recommendations for the public (6.2%), and quarantine (5.9%) (Basch et al., 2020). However, racial minorities were underrepresented in COVID-19 news articles (January 21 through June, 2020) (Xu et al., 2022). Another study revealed that the coverage of African American issues (between March and May 15, 2020) by Black websites and mainstream news outlets were very similar to each other, including emphasizing societal over individual causes of racial disparities in health outcomes (Biswas et al., 2021). Evaluating the characteristics of messaging on COVID-19-related racial disparities is essential in understanding the role that local TV news may have played in shaping public knowledge regarding COVID-19 and related racial disparities. It may also offer guidance for strategic efforts to improve this coverage in ways that better inform Americans and invite support for policies that address the social and structural factors that cause racial health disparities in the first place.

### The Murder of George Floyd and the Potential Impact on Coverage of Racial Disparities

This study, unlike previous studies, also looks at coverage before and after the murder of George Floyd, which gained nationwide attention in the days and weeks that followed. On May 25, 2020, an African American man named George Perry Floyd Jr. was murdered by a White police officer in Minneapolis, Minnesota. The murder, captured by onlookers on mobile phone cameras, happened during the arrest after a store clerk suspected Floyd may have used a counterfeit $20 bill. We look at this event for two reasons. On the one hand, major US newspapers covered the murder and subsequent protests and uprisings extensively throughout late May and early June (Reny & Newman, 2021). Given the inherent limitations of broadcast time and news media attention, we reasoned that coverage of protests for racial justice could have displaced local TV news coverage about COVID-19 in general, and racial disparities in COVID-19 outcomes more specifically. On the other hand, the murder and national movement that followed may have also shined a light on systemic racism and racial injustice/inequity in the context of COVID-19. For example, one group of researchers analyzed posts on Twitter and found that public discourse showed both a decline in negative sentiments toward Black people after the murder of George Floyd, and an increase in awareness of structural racism and desire for social change (Nguyen et al., 2021). Prior research has also found that Black-led racial justice protests can influence news media coverage: non-violent protests can lead to positive media coverage and benefit the larger civil rights movement, while violent protests can lead to negative media coverage and hurt the movement (Wasow, 2020). Because the murder of George Floyd and the subsequent racial justice protests overlapped with the early stage of the COVID-19 pandemic, it seems plausible that the murder and national movement could have promoted increased media attention to racial disparities in COVID-19. These competing possibilities both highlight the need to understand whether local TV news coverage about COVID-19-related racial disparities differed before and after George Floyd’s murder.

This research builds on an emerging body of work on news coverage and media framing of health disparities and social determinants of health. We conducted a quantitative content analysis to examine the coverage and framing of COVID-19-related racial/ethnic disparities in physical health and social outcomes in local evening TV news stories during the first wave of the COVID-19 pandemic (March to June 2020). We focused particular attention on how local news stories attributed causes and solutions for COVID-19-related health disparities between racial groups and how coverage of COVID-19-related racial disparities changed over time.

### Research Questions

We ask two broad research questions (RQs) built on literature about framing and news content analysis in the context of health disparities and social determinants of health:

**RQ1:** In news stories that reference COVID-19-related health disparities in health or social outcomes, to what extent do stories attribute those differences to individual factors or to larger structural factors?

**RQ2:** How did local TV news coverage of racial disparities related to COVID-19 differ before and after the murder of George Floyd?

## Methods

We first selected 22 media markets out of 210 total markets in the U.S. and selected a cohort of 85 stations. We obtained video clips and transcripts from TV Eyes (https://tveyes.com/), a global search engine for TV and radio coverage. We focused on a single half-hour window of each late-evening local TV news broadcast (usually 10:00–10:30 pm or 11:00–11:30 pm) from each of the four major broadcast networks (ABC, CBS, FOX, NBC) that have affiliates within a particular market (most have all four, though some have 2 or 3), for a total of 85 stations tracked in the 22 media markets. The 22 media markets selected for analysis included: Albuquerque, NM; Bangor, ME; Casper, WY; Cincinnati, OH; Columbia, SC; Detroit, MI; Flint, MI; Greenville, SC; Hartford, CT; Houston, TX; Miami, FL; Milwaukee, WI; Minneapolis, MN; Mobile, AL; New York, NY; Phoenix, AZ; Portland, ME; Syracuse, NY; Tampa, FL; Wichita, KS; Youngstown, OH; Seattle, WA. We chose these markets strategically for variation in (a) COVID-19 cases, hospitalizations, or deaths, (b) demographic factors, and (c) political ideology. First, we chose markets in geographically diverse locations with high COVID-19 severity (case and death rates) in the early phase of the pandemic, including New York, NY, Seattle, WA, and Houston, TX. We also included Albuquerque, NM, and Phoenix, AZ, both of which experienced severe COVID-19 outbreaks and include large Indigenous populations known to have had a disproportionately large COVID-19 burden (for instance, the Navajo Nation). We included Minneapolis, MN because it was where the murder of George Floyd happened. We also selected some smaller markets with relatively low reported COVID-19 cases, including Portland, ME, Bangor, ME, and Casper, WY. In addition, we sought to include geographically diverse markets with additional variability in overall political orientation, as measured by the percentage of Trump vote share (in the 2016 election): Southern markets: Greenville, SC (conservative: 67% Trump vote share), Columbia, SC (balanced: 46%), Mobile, AL (conservative: 67%), and Miami, FL (liberal: 37%); Midwestern markets: Detroit, MI (liberal leaning: 42%), Flint, MI (balanced: 51%), Wichita, KS (conservative: 66%), Milwaukee, WI (liberal leaning: 43%), Cincinnati, OH (conservative leaning: 59%), and Youngstown, OH (balanced: 53%); and other Northeastern markets: Hartford, CT (liberal leaning: 44%) and Syracuse, NY (liberal leaning: 45%).

### Keyword Validation and Searching

We began the process of identifying content focused on COVID-19 and racial disparities through an iterative and systematic search term validation procedure (Stryker et al., 2016). We conducted keyword searches of broadcast transcripts produced for closed captioning purposes. We chose a random set of dates within the observation period for the search term validation procedure that would not be sampled in the formal content analysis that followed. We sought to maximize recall (how well the search phrases accurately call up all relevant items) while achieving a reasonable precision (whether the search phrases can avoid calling up irrelevant items) because candidate news clips would be screened further by human coders and thus could correct for limited keyword search result precision but not recall. The human coding process cannot account for errors of omission (hence the need to maximize recall), but it can screen irrelevant items in later stages of the coding process (i.e., human coders would have the opportunity to judge a clip as irrelevant and discard it from the sample). This approach identified relevant clips with a recall of 91% (i.e., the search term caught 91% of relevant stories) and a precision of 38% (i.e., 38% of stories captured by the search term were indeed relevant). Specifically, we used 100 human coded clips to validate our keywords, among which 11 news stories were identified as relevant by human coders. By using keywords developed by our team, the machine retrieved 26 stories in total. Ten out of the 26 stories matched relevant racial disparities news stories identified by human coders (91% recall = 10 machine retrieved relevant stories/11 human coded relevant stories), and 16 were deemed as irrelevant news stories (38% precision = 10 machine retrieved relevant stories/26 total stories retrieved by machine). We missed one relevant clip in the validation round due to transcription error, and some irrelevant stories were captured because they referenced black as the color of objects (e.g., black bear). We prioritized high recall over high precision for the search term itself because we proceeded to a second phase of human coding in which we were able to further screen all the stories identified by the search term with human coders and screen the irrelevant ones out.

The final search term included a combination of least one term denoting a particular racial or ethnic group (“racial disparity”, “racial disparities”, “racism”, “minority”, “minorities”, “people of color”, “community of color”, “communities of color”, “black”, “african american”, “hispanic”, “latino”, “latinx”, “latina”, “asian american”, “indigenous”, “american indian”, “native american”, “hawaiian and pacific islanders”, “alaska native”, “navajo”, “choctaw”, “apache”, “pueblo of zia”, “pueblo of san felipe”, “kewa pueblo”, “winnebago”, “colorado river indian reservation”, “pueblo of zuni”, “ho chunk”, “lummi”, “cherokee”, “indian reserve”, “indian reservation”) and at least one COVID-19 related keyword (“corona”, “covid”, “coronavirus”, “virus”, “quarantine”, “stay at home”, “shelter in place”, “shelter at home”, “mask”, “symptomatic”, “symptom”, “negative”, “positive”, “pandemic”, with the exclusion of “black smoke”, “race against time”, “cherokee boulevard”).

### Sampling of a Constructed Week Per Month

We then limited the sample to just the news hits that aired on dates within the “constructed weeks”: For each month between March and June 2020, we selected one weekday at random (e.g., one Monday, one Tuesday) to create what is called a “constructed week” sample of news coverage (20 sampled days out of 87 total weekdays), a method validated in previous media research to generate representative news content (Luke et al., 2011). This process yielded an analytic sample of 918 keyword search hits.

### News Story Segment Clipping

The next step in the data collection process was to clip the news story (assign a start and end time for discrete news segments) identified by the hits. Trained research assistants watched the video surrounding all 918 keyword search term hits and identified story start and stop times based on both visual and audio cues (e.g., a change of news topic or change of reporter). Twelve research assistants clipped all news stories, and each hit was double clipped by a pair of research assistants to ensure that coders were in agreement about the start and end time for each clip.

### Relevance Identification

For a clipped news story to be classified as relevant, (1) it had to mention race/ethnicity in the context of a COVID-19-related impact for either people living in the United States or US citizens, and (2) it could not be a duplicate of an already identified story. We excluded a substantial number of hits that coders identified as duplicates (*n* = 391) or not being relevant stories (*n* = 358), leaving an analytic sample of 169 identified relevant stories. Coders were highly reliable in this assessment (Krippendorff’s *α* = 0.94).

### Content Coding Instrument and Measures

We developed a coding instrument to capture information about COVID-19-related racial disparities in local news through an iterative process that included (a) multiple conversations with the full research team to identify categories of potentially salient content, and (b) multiple inductive analyses of local TV news stories about COVID-19-related racial disparities from dates outside of the study’s sampling frame. Coders first assessed whether the story concerned mainly a specific physical/mental or societal outcome of COVID-19 that is linked to a specific racial/ethnic group. We examined different types of outcomes for (a) physical and mental health disparities, and for (b) societal disparities. We then examined causal explanations of and potential solutions for these disparities. For both causes and solutions, we distinguished between those that focused on individual behaviors/conditions (e.g., pre-existing conditions, risky behaviors) versus those that focused on broader social factors (e.g., medical access, workplace safety). Even though pre-existing conditions are also outcomes of social inequality, we describe them as individual causal explanations because news stories about COVID-19 rarely described pre-existing conditions as stemming from structural causes, and prior research overwhelmingly indicates that people attribute health outcomes such as obesity, diabetes, heart disease, and many cancers to individual decisions and behaviors (Robert & Booske, 2011; Vishwanath, 2014; Wolfson et al., 2015). We then coded for specific references to bias incidents and instances of racial discrimination.

### Coding Process and Interrater Reliability

Each of six trained coders analyzed the same 80 training clips in sets of 15–20 until Krippendorff’s *α* averaged > 0.80. We then distributed clips from the analytic sample among all six coders, and each clip was coded independently by at least one coder. To assess interrater reliability (see Tables 1, 2 and 3), 20% of the clips (*n* = 110) were double coded by two different coders. We used all 110 clips to assess interrater reliability for screening variables, e.g., clip type and relevance judgment. For all other variables regarding story details, we only used relevant clips (*n* = 81) to assess interrater reliability. For each of these double-coded clips, one coder’s codes were randomly chosen for the final analytic dataset. Coding assignments were completed between June 2021 and August 2021.

**Table 1:** Descriptive data on the frequency, inter-rater reliability, and relative prevalence of COVID-19 local TV news stories overall and pre- versus post-George Floyd’s murder

	Freq	Pct. (%)^a^	Alpha	% Diff pre-post Floyd^b^	χ^2^	*P*
*Clip type*			0.91		2.02	0.16
Local news	158	93		6.4	–	–
Teaser	11	7		− 6.4	–	–
*Focus (versus mention)*	114	67	1.00	− 10.6	2.22	0.14
*Population impacted*						
Indigenous people are impacted	70	41	1.00	− 21.9	3.57	0.06
Black people are impacted	64	38	0.91	22.9	7.81	< 0.01
Minorities/POC are impacted	42	25	1.00	10.2	1.84	0.18
Latinx people are impacted	22	13	1.00	7.1	4.18	0.04
Asian people are impacted	15	9	1.00	− 4.1	0.07	0.79
White people are impacted	14	8	1.00	5.3	1.22	0.27
Pacific Islanders are impacted	1	1	1.00	− 0.8	0.00	1.00
*Physical illness outcomes mentioned*	131	78	0.75	− 2.2	0.00	0.97
*Mental health outcomes mentioned*	1	1	1.00	2.0	0.13	0.72
*Societal impacts mentioned*	29	17	0.88	− 4.5	0.84	0.36
*Racial justice protestors mentioned*	8	5	1.00	16.0	13.9	0.00

^a^The denominator is 169, the total number of relevant news stories

^b^The column “diff. pre-post-George Floyd” presents the percentage point change in the percentage of stories that reference the topic before and after George Floyd’s murder on May 25, 2020

### Analytic Approach

We calculated descriptive statistics for the aforementioned variables relevant to news coverage of COVID-19-related racial disparities. We also calculated the difference (in percentage points) for variable frequencies pre- and post-George Floyd’s murder and conducted Chi-square tests to identify whether those differences were statistically significant.

## Results

**Fig. 1:**
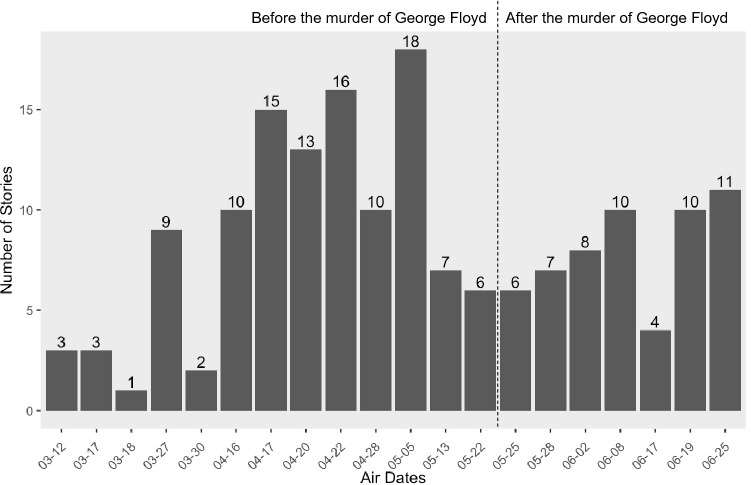
Distribution of news stories from March 12, 2020 to June 25, 2020

Across the 85 stations sampled for 20 days, there was an average of 8.45 relevant news stories per day. Figure 1 provides an overview of distribution of news stories from March 12, 2020 to June 25, 2020.

Most (93%, *n* = 158) of the 169 stories in the sample were full stories, whereas 7% (*n* = 11) were teasers (short previews of upcoming news stories; see Table 1). Roughly a third (33%, *n* = 55) of news stories contained a passing mention of race/ethnicity and a COVID-19-related impact, while a majority (67%, *n* = 114) were the primary focus of the news segment. Figure 1 shows the distribution of news stories over time. The overall volume was roughly equivalent before (8.69 stories/day) and in the immediate aftermath (8.00 stories/day) of the murder of George Floyd on May 25, 2020. About 29% (*n* = 49) of the 169 stories were from Albuquerque, New Mexico, which were mainly about Indigenous members of the Navajo Nation.

### Types of Outcomes with Racial Disparities and Impacted Racial/Ethnic Groups

Most (78%, *n* = 131) of the 169 stories were about physical illness outcomes, with only 1% (*n* = 1) about mental health outcomes. Most outcomes were about COVID-19 cases (69%, *n* = 117), including infection, rates (of infection), or non-specific mentions of COVID impact (e.g., “the Navajo Nation was hit hard by COVID-19”). Another third (33%, *n* = 56) of stories were about deaths due to COVID-19. Very few news stories mentioned hospitalizations (1%, *n* = 1) and being on ventilators (1%, *n* = 1).

About 17% (*n* = 29) stories were societal-related racial disparities, and the most common outcome discussed was racial discrimination (8%, *n* = 13), followed by employment/income (7%, *n* = 11). A few stories mentioned exposure to policing for COVID-19-related infractions, such as avoiding masks or not practicing social distancing (7%, *n* = 11), and one mentioned housing insecurity (1%, *n* = 1).

Most stories focused on racial/ethnic health disparities affecting either Native Americans (41%, *n* = 70), African Americans (38%, *n* = 64), and non-specific mention of people of color (25%, *n* = 42). Relatively few stories focused on Hispanic/Latinx people (13%, *n* = 22), Asian people (9%, *n* = 15), or Pacific Islanders (1%, *n* = 1). About 8% of stories mentioned White people (*n* = 14), but mostly as a reference group (e.g., “Black individuals are more likely to get COVID-19 than white individuals”).

### Causal Explanations

Among the 132 stories that mentioned physical/mental outcomes, 42 stories (32%) offered causal explanations. Most stories mentioned at least one structural factor (95% of the 42 stories, *n* = 40; See Table 2), including medical access (31% of the 42 stories with causal attributions, *n* = 13), testing (29%, *n* = 12), close quarters (17%, *n* = 7), economic hardship (17%, *n* = 7), general structural issues (14%, *n* = 6), transportation (12%, *n* = 5), workplace safety (10%, *n* = 4), food insecurity (10%, *n* = 4), lack of resources (10%, *n* = 4), access to masks (5%, *n* = 2), access to handwashing (5%, *n* = 2), education (5%, *n* = 2), racism (5%, *n* = 2), housing (2%, *n* = 1), and access to PPE (2%, *n* = 1). Very few causal explanations focused on individual explanations (17% of the 42 stories, *n* = 7), including pre-existing conditions (14%, *n* = 6) and risky behaviors such as attending parties (2%, *n* = 1). Stories were categorized as undetermined if they could be grouped as either structural or individual causes, including social distancing (24%, *n* = 10). Two-thirds of the 132 stories (*n* = 90) provided no cause at all.

**Table 2:** Descriptive data on the frequency, inter-rater reliability, and relative prevalence of causal and solution attributions in COVID-19 local TV news stories, overall and pre- versus post-George Floyd’s murder

	Freq	Pct. (%)^a^	Alpha	% Diff pre-post Floyd^b^	χ^2^	*P*
*Physical outcomes*						
Cases as physical outcome	117	69	0.68	− 1.7	0.01	0.92
Deaths as physical outcome	56	33	0.88	− 27.2	6.00	0.01
Write-in physical outcome	2	1	1.00	4.0	1.60	0.21
Hospitalizations as physical outcome	1	1	1.00	− 0.8	0.13	0.72
Ventilator as physical outcome	1	1	1.00	2.0	0.13	0.72
Emergency as physical outcome	0	0	1.00	0.0	–	–
ICU as physical outcome	0	0	1.00	0.0	–	–
Long haulers as physical outcome	0	0	1.00	0.0	–	–
Risk behaviors as physical outcome	0	0	1.00	0.0	–	–
Other disease as physical outcome	0	0	1.00	0.0	–	–
*Mental health outcomes*						
Nonclinical mental health outcome	1	1	1.00	2.0	0.13	0.72
Clinical mental health outcome	0	0	1.00	0.0	–	–
Causes of physical/mental impacts						
*Individual level causes*	7	4	1.00	− 0.2	0.02	0.88
Preexisting conditions as physical/mental cause	6	4	1.00	0.6	0.00	1.00
Risky behaviors as physical/mental cause	1	1	1.00	− 0.8	0.13	0.72
*Structural level causes*	40	24	0.78	6.2	0.75	0.39
Medical access as physical/mental cause	13	8	1.00	− 2.4	0.00	1.00
Testing as physical/mental cause	12	7	0.62	1.3	0.00	1.00
Close quarters as physical/mental cause	7	4	1.00	− 0.2	0.00	1.00
Economic hardship as physical/mental cause	7	4	0.00	− 0.2	0.02	0.88
Structural issues as physical/mental cause	6	4	0.65	9.2	4.92	0.03
Transportation as physical/mental cause	5	3	0.00	− 1.4	0.02	0.88
Workplace safety as physical/mental cause	4	2	0.65	− 0.5	0.04	0.85
Food insecurity as physical/mental cause	4	2	1.00	− 0.5	0.00	1.00
Lack of resources as physical/mental cause	4	2	1.00	2.3	0.04	0.85
Masks as physical/mental cause	2	1	1.00	4.0	1.6	0.21
Handwashing as physical/mental cause	2	1	1.00	− 1.7	0.06	0.81
Education as physical/mental cause	2	1	1.00	− 1.7	0.06	0.81
Racism as physical/mental cause	2	1	1.00	4.0	1.60	0.21
Housing as physical/mental cause	1	1	0.00	− 0.8	0.00	1.00
PPE access as physical/mental cause	1	1	1.00	− 0.8	0.00	1.00
Incarceration as physical/mental cause	0	0	1.00	0.0	–	–
*Undetermined level causes*						
Social distancing as physical/mental cause	10	6	1.00	3.0	0.02	0.90
Physical/mental solutions						
*Individual level solutions*			1.00			
Behavioral intervention as physical/mental solution	8	5	1.00	1.8	0.00	1.00
*Structural level solutions*	50	30	0.795	− 2.3	0.15	0.70
Community resources, federal government help, or economic resources as physical/mental solution	28	17	0.776	− 6.5	0.61	0.43
Lockdown as physical/mental solution	18	11	0.779	− 3.8	0.6	0.44
PPE distribution as physical/mental solution	10	6	0.779	− 8.4	1.58	0.21
Medical access as physical/mental solution	5	3	1.00	7.2	3.16	0.08
Medical distribution as physical/mental solution	3	2	0.00	− 2.5	0.37	0.54
Racial justice as physical/mental solution	2	1	1.00	4.0	1.6	0.21
Clinical mental health as physical/mental solution	1	1	1.00	2.0	0.13	0.72
Workplace safety as physical/mental solution	0	0	1.00	0.0	–	–
Transportation as physical/mental solution	0	0	1.00	0.0	–	–
*Undetermined level solutions*						
Testing as physical/mental solution	33	20	1.00	− 10.7	3.34	0.07
Other physical/mental solution	21	12	0.104	5.1	0.58	0.44
*Preexisting conditions mentioned*						
Diabetes as pre-existing condition	5	3	1.00	− 1.4	0.02	0.88
Hypertension as pre-existing condition	4	2	1.00	2.3	0.04	0.85
Lung diseases as pre-existing condition	1	1	1.00	− 0.8	0.00	1.00
Obesity as pre-existing condition	1	1	1.00	2.0	0.13	0.72
Other pre-existing condition	1	1	1.00	− 0.8	0.00	1.00
*Societal outcomes mentioned*						
Discrimination as societal outcome	13	8	1.00	− 5.2	1.23	0.27
Employment/income as societal outcome	11	7	0.83	− 0.7	0.01	0.92
Housing as societal outcome	1	1	1.00	2.0	0.13	0.72
Policing as societal outcome	1	1	1.00	− 0.8	0.00	1.00
Causes of societal impacts						
*Structural level causes*	14	8	0.78	− 0.4	0.01	0.93
Racism as societal cause	9	5	1.00	− 4.7	1.16	0.28
Economic hardship as societal cause	5	3	0.78	4.3	0.66	0.42
Blaming China as societal cause	4	2	1.00	− 3.4	0.79	0.38
Lack of resources as societal cause	1	1	1.00	2.0	0.13	0.72
Societal solutions						
*Structural level solutions*	9	5	0.65	− 1.9	0.12	0.73
Economic resources as societal solution	7	4	0.65	− 3.0	0.45	0.50
Community resources as societal solution	1	1	1.00	− 0.8	0.00	1.00
Address racism as societal solution	1	1	1.00	2.0	0.13	0.72
Racial justice as societal solution	1	1	1.00	2.0	0.13	0.72
Federal government help as societal solution	1	1	1.00	− 0.8	0.00	1.00
*Discrimination details*						
Broad pattern of discrimination mentioned	14	8	1.00	− 3.2	0.46	0.50
Specific incident of discrimination mentioned	8	5	1.00	− 3.9	0.78	0.38
*Action taken to eliminate discrimination*						
Police investigation discrimination	4	2	1.00	− 0.5	0.00	1.00
Non-police action to reduce discrimination	3	2	1.00	0.3	0.00	1.00
Legislative action to reduce discrimination	1	1	1.00	2.0	0.13	0.72
*People voicing support for victims of bias incidents*						
Local leaders are supportive of victims of bias incident	5	3	1.00	− 1.4	0.02	0.88
Governor is supportive of victims of bias incident	1	1	1.00	2.0	0.13	0.72
Trump is supportive of victims of bias incident	0	0	1.00	0.0	–	–
Legislator is supportive of victims of bias incident	0	0	1.00	0.0	–	–
*Solutions to societal impacts*						
Other societal solution	2	1	0.00	1.2	0.00	1.00
Call for support as societal solution	1	1	1.00	2.0	0.13	0.72
Testing as societal solution	1	1	1.00	− 0.8	0.00	1.00
Workplace safety as societal solution	1	1	1.00	− 0.8	0.00	1.00

^a^The denominator is 169, the total number of relevant news stories

^b^The column “diff. pre-post-George Floyd” presents the percentage point change in the percentage of stories that reference the topic before and after George Floyd’s murder on May 25, 2020

Among the 29 stories that included societal outcomes, 14 stories provided causal explanations, which were structural in focus (100% of the 14 stories, *n* = 14), including racism (64%, *n* = 9), economic hardship (36%, *n* = 5), blaming China (29%, *n* = 4), and lack of resources (7%, *n* = 1). More than half of the 29 stories (*n* = 15) provided no cause at all.

### Solution Explanations

Among the 132 stories that mentioned physical and mental impacts, 86 stories (65%) mentioned potential solutions, many were structural/policy oriented in nature (58% of the 86 stories, *n* = 50), including lockdowns (11%, *n* = 18), provision of federal government help, community, or economic resources (17%, *n* = 28), and PPE distribution (6%, *n* = 10). Fewer stories focused on increased medical access (3%, *n* = 5), medical distribution (2%, *n* = 3), racial justice (1%, *n* = 2), or clinical mental health care (1%, *n* = 1). Many stories mentioned testing (20%, *n* = 33), which we classified as an undetermined solution because these stories could have emphasized either testing availability or individual decisions to get tested when symptomatic or at risk. Very few stories focused on behavioral interventions as an individual-focused solution (5% of the 86 stories, *n* = 8). A little over a third of the 132 stories (*n* = 46) provided no solution entirely.

Among the 29 stories that mentioned societal outcomes, nine (31%) stories described potential solutions, with structural solutions in focus (100% of the 9 stories, *n* = 9). These included provision of economic resources (78% out of 9, *n* = 7), community resources (11%, *n* = 1), addressing racism (11%, *n* = 1), racial justice (11%, *n* = 1), and federal government help (11%, *n* = 1). Around two-thirds of the 29 stories (*n* = 20) provided no solution entirely.

### Racial Discrimination Incidents

Racial discrimination stories took the form of (a) systematic discussions of broad patterns of discrimination (8% of the 169 stories, *n* = 14) and (b) specific episodic bias/discrimination incidents (5%, *n* = 8). To address discrimination, stories focused on police investigation (2%, *n* = 4), non-police action (2%, *n* = 3), and legislative action (1%, *n* = 1). News stories occasionally included governors (1%, *n* = 1) and local leaders (3%, *n* = 5) speaking in support of victims of incidents of discrimination.

### Additional Story Details

Very few stories included mentions of research or studies (5% of 169 stories, *n* = 8; see Table 3). A substantial number, however, presented statistics or visual data depictions (27%, *n* = 46).

**Table 3:** Descriptive data on the frequency, inter-rater reliability, and relative prevalence of research, sources, and references to racial justice and racism in local TV news stories, overall and pre- versus post-George Floyd’s murder

	Freq	Pct. (%)^a^	Alpha	% Diff pre-post Floyd^b^	χ^2^	*P*
Mention of research	8	5	1.00	1.8	0.00	1.00
Data or graphs displayed	46	27	0.81	− 15.9	6.13	0.01
Location mentioned	150	89	0.65	− 15.3	7.25	< 0.01
Nation mentioned	23	14	− 0.05	0.6	0.00	1.00
State mentioned	63	37	0.51	− 7.5	0.64	0.42
Local mentioned	71	42	0.73	0.0	–	–
Tribal mentioned	62	37	1.00	− 23.7	5.71	0.02
Location comparison	25	15	0.75	− 4.0	0.67	0.41
People mentioned						
President Trump mentioned or pictured or heard speaking	8	5	0.65	− 3.9	0.78	0.376
Surgeon General mentioned or pictured or heard speaking	2	1	1.00	4	1.60	0.206
Democrats mentioned or pictured or heard speaking	18	11	1.00	− 5.8	0.60	0.438
Republicans mentioned or pictured or heard speaking	2	1	1.00	4	1.60	0.206
Politician mentioned or pictured or heard speaking	65	38	0.71	5.9	0.99	0.320
Health representative mentioned or pictured or heard speaking	28	17	0.57	5.7	0.95	0.329
Researcher mentioned or pictured or heard speaking	3	2	1.00	− 2.5	0.37	0.541
Police official mentioned or pictured or heard speaking	2	1	1.00	− 1.7	0.06	0.806
Exemplar						
Personal exemplar	43	25	0.80	3.6	0.00	1.00
Exemplar visualized	39	23	0.80	7.0	0.05	0.82
Racial justice movement reference	15	9	1.00	30.0	30.0	< 0.01
Mention of “racism”	16	9	1.00	3.6	0.01	0.91

^a^The denominator is 169, the total number of relevant news stories

^b^The column “diff. pre-post-George Floyd” presents the percentage point change in the percentage of stories that reference the topic before and after George Floyd’s murder on May 25, 2020

Politicians were the most referenced spokespeople or source (i.e., mentioned/pictured/heard speaking) in local TV news stories describing racial disparities in COVID-19-related outcomes (38% of 169 stories, *n* = 65), followed by health experts (17%, *n* = 28). Relatively few stories referenced researchers (2%, *n* = 3), the Surgeon General (1%, *n* = 2), or police officials (1%, *n* = 2). Among politicians, references to Democrats (11%, *n* = 18) were far more common than Republicans (1%, *n* = 2). The references to health experts should be interpreted with some caution due to the reliability 0.57.

A quarter of stories (25%) included personal exemplars (e.g., people experiencing COVID-related impacts, *n* = 43). In 91% of those 43 stories, the exemplar was visualized (*n* = 39).

Finally, only a handful of stories included explicit mentions of “racism” (9% of 169 stories, *n* = 16) and a nearly equal number of stories involved references to the racial justice movement (9%, *n* = 15). A small number of stories also mentioned protestors for racial justice in a way that connected directly to COVID-related impacts on a particular racial/ethnic group (5%, *n* = 8).

### Differences in Patterns of Coverage Before and After the Murder of George Floyd

Most variables we examined were similar in frequency before and after the murder of George Floyd. However, there were some notable differences for variables regarding race and racial justice.

After the murder, mentions of impacted African Americans (+ 22.9%, *χ*^2^(1) = 7.81, *p* < 0.01) and Hispanic/Latinx people (+ 7.1%, *χ*^2^(1) = 4.18, *p* = 0.04) increased relative to earlier weeks. There was also a trending, though non-significant decline in the percentage of stories that referenced impacts on Indigenous people (− 21.9%, *χ*^2^(1) = 3.57, *p* = 0.06) and fewer mentions of tribal nations (− 23.7%, *χ*^2^(1) = 5.71, *p* = 0.02).

After Floyd’s murder, there were fewer mentions of deaths due to COVID-19, *χ*^2^(1) = 6.00, *p* = 0.014 (− 27.2%), which might be related to the fact that COVID-19-related death rates dropped precipitously during May and June. For physical/mental outcomes, there were more mentions of causal explanations related to structural inequality and discrimination, *χ*^2^(1) = 4.92, *p* = 0.027 (+ 9.2%). There were fewer displays of data points or graphs/charts on screen that described COVID-19-related impacts on a particular racial/ethnic group, after the murder, *χ*^2^(1) = 6.13, *p* = 0.013 (− 15.9%). There were more mentions of COVID-19-related impacts on people protesting for racial justice, *χ*^2^(1) = 13.93, *p* < 0.001 (+ 16.0%), as well as more reference to the racial justice movement overall, *χ*^2^(1) = 29.99, *p* < 0.001 (+ 30.0%).

## Discussion

Our analysis, combined with similar findings from Biswas et al. (2021), suggests that structural (versus individual) explanations were more discussed in news of COVID-19 impacts on people of color in the early stages of the pandemic in the U.S. This is contrary to other previous studies of racial/ethnic disparities in non-COVID health outcomes (Nagler et al., 2016; Taylor-Clark et al., 2007). For instance, Kim et al (2010) found that only 30% of newspaper articles from 1996 to 2005 provided causal or solution explanations for health disparities, and behavioral (vs societal/health care) explanations dominated the discourse. Meanwhile, around 62% of the stories (*n* = 90 for physical/mental; *n* = 15 for societal) did not offer a causal explanation at all. Unfortunately, scholars established that people default to individual explanations for health outcomes in the absence of information that attributes causes and solutions to larger structural factors (Gilens, 2000; Gilliam & Iyengar, 2000; Robert & Booske, 2011), suggesting that much of the coverage missed an opportunity to bring wider public awareness to their social determinants. Public health advocates and journalists should seek to imbue local TV news stories with more frequent discussion about causes and solutions of these disparities, especially explanations from a structural perspective to raise public awareness of health disparities (Kim et al., 2010; Niederdeppe et al., 2008). Our findings contribute to the broader literature on emphasis framing, and the more specific literature on framing of racial health disparities in the media, by examining how attribution and responsibility frames are presented in local TV news media. Future research should also examine how attribution and responsibility frames can influence the ways that audiences attribute the causes of, and solutions to, COVID-19-related racial disparities in health and social outcomes.

While none of the information sources we examined were very common in the stories, references to politicians were more common than health experts, and Democrats more than Republicans. Similarly, an earlier study found that during the initial stage of the pandemic, newspaper coverage mentioned politicians more frequently than scientists (Hart et al., 2020). These patterns of coverage may have contributed to what have become heavily politicized debates about COVID-19 mitigation strategies in general and racial justice more broadly (Stroebe et al., 2021). When health issues become increasingly politicized, the public will decrease their support toward health requirements/programs and their confidence in doctors and governments, which will have long-lasting implications (Fowler & Gollust, 2015).

Only 9% of the 169 stories referenced racism, and there was no significant difference in the frequency of such mentions before and after the murder of George Floyd. This is consistent with the pattern Kim et al. (2010) found in their analysis of newspaper articles in the 90 s and 00 s: only 4% of newspaper articles they analyzed invoked a social-justice rationale. Two recent studies also found that scientific publications have relatively few references to the word racism (Hardeman et al., 2018; Krieger et al., 2021). Despite the rising incidence of scholarly work describing racism as a public health crisis (Andrews, 2021), the early phases of the pandemic did not regularly employ such a frame. Systemic racism and structural discrimination are primary determinants of racial health disparities in the US (Feagin & Bennefield, 2014). There is a dire need for the public health community to call greater attention to racism’s role in producing and maintaining health disparities in the US, particularly when researchers and advocates have the opportunity to speak with news outlets (Orr et al., 2021). Yet at the same time, more research is needed about both when and how media coverage should emphasize racism in coverage (Kilgo, 2021), and the impact of the explicit linkages between race and health on the public’s attitudes and opinions (Gollust et al., 2022).

### Limitations

First, our sample is not representative of all local TV news coverage because we purposefully selected media markets for variability in a variety of COVID-related and aggregate demographic characteristics. Second, this content analysis only includes local broadcast TV news, so our results may not be generalizable to other sources of news coverage. Third, like most content analysis projects, there is some subjectivity during the process of coding local news. However, such subjectivity is not likely to be a major concern because the interrater reliability was greater than Krippendorff’s alpha of 0.70 for most coded measures included. Fourth, while achieving very high recall (91%), we cannot rule out the possibility that the 9% error could have led to systematic differences in the types of stories retrieved by the search term. We believe this unlikely, however, based on the rigor of the search term calibration and validation process.

## Conclusions

This study provides evidence that social determinants of COVID-19 related racial disparities have been part of the discussion in local TV news. When stories offered explanations, they were more likely to offer structural ones. However, most stories described COVID-19 disparities without any causal explanations, and local TV news rarely attributed these disparities to racism or racial discrimination. Future research should examine media coverage patterns for COVID-19 or other health-related racial disparities from other news sources. We also need to better understand audience’s interpretations of these causal and solution explanations, and how local news programs and health advocates/practitioners can design effective messages to raise awareness about social determinants and structural explanations of health disparities.
